# Investigation of Childhood Lead Poisoning from Parental Take-Home Exposure from an Electronic Scrap Recycling Facility — Ohio, 2012

**Published:** 2015-07-17

**Authors:** Nick Newman, Camille Jones, Elena Page, Diana Ceballos, Aalok Oza

**Affiliations:** 1 Cincinnati Children’s Hospital Pediatric Environmental Health Specialty Unit; 2 City of Cincinnati Health Department Childhood Lead Poisoning Prevention Program; 3 Division of Surveillance, Hazard Evaluations, and Field Studies, National Institute for Occupational Safety and Health, CDC

Lead affects the developing nervous system of children, and no safe blood lead level (BLL) in children has been identified (1). Elevated BLLs in childhood are associated with hyperactivity, attention problems, conduct problems, and impairment in cognition (2). Young children are at higher risk for environmental lead exposure from putting their hands or contaminated objects in their mouth. Although deteriorating lead paint in pre-1979 housing is the most common source of lead exposure in children, data indicate that ≥30% of children with elevated BLLs were exposed through a source other than paint (3). Take-home contamination occurs when lead dust is transferred from the workplace on employees’ skin, clothing, shoes, and other personal items to their car and home (4). Recycling of used electronics (e-scrap) is a relatively recent source of exposure to developmental neurotoxicants, including lead (5). In 2010, the Cincinnati Health Department and Cincinnati Children’s Hospital Pediatric Environmental Health Specialty Unit (PEHSU) investigated two cases of childhood lead poisoning in a single family. In 2012, CDC’s National Institute for Occupational Safety and Health (NIOSH) learned about the lead poisonings during an evaluation of the e-scrap recycling facility where the father of the two children with lead poisoning worked. This report summarizes the case investigation. Pediatricians should ask about parents’ occupations and hobbies that might involve lead when evaluating elevated BLLs in children, in routine lead screening questionnaires, and in evaluating children with signs or symptoms of lead exposure.

In June 2010, a male child aged 1 year and a female child aged 2 years were identified by routine screening to have elevated BLLs of 18 *μ*g/dL and 14 *μ*g/dL, respectively. The children’s primary care physician referred them to the Cincinnati Children’s Hospital PEHSU, and the Cincinnati Health Department’s Childhood Lead Poisoning Prevention Program completed a lead risk assessment at the family’s home. The father worked at an e-scrap recycler company (facility A), crushing cathode ray tubes. He did not wear personal protective equipment at work, and he reported playing with his children when he came home. The family reported there was frequently visible dust in his hair, and the children often touched his hair. The father’s BLL was 25 *μ*g/dL. The lead risk assessment revealed detectable lead dust on the floor of the home, but no lead-containing paint was detected in the home. The children attended daycare in a building that was built in 1992. The father was advised to notify the Occupational Safety and Health Administration of his BLL; it is not known if he did. The father left his job soon after the elevated BLLs were recognized, and the children’s BLLs decreased to 8.7 *μ*g/dL and 7.9 *μ*g/dL, respectively, over the next 3 months.

In 2012, in an activity unrelated to the lead poisoning incident described in this report, NIOSH conducted a health hazard evaluation at facility A, as part of an initiative to learn more about exposures in e-scrap recycling. NIOSH was unaware of the childhood lead poisonings, as was the employer. The PEHSU investigator became aware of the NIOSH evaluation through a notification to a local affiliated occupational medicine training program and contacted the NIOSH investigators to notify them.

NIOSH investigators performed air and surface sampling for lead throughout facility A, which employed approximately 80 persons. Three wipe samples taken from work surfaces in the cathode ray tubes area indicated high levels of lead. Cathode ray tubes are made from leaded glass, with lead concentrations in the funnel glass up to 25% and in the frit (where the panel glass joins the funnel glass) up to 85%. Lower surface lead concentrations were found outside the production area, including in the conference room supply air duct, multiple places in the break room (e.g., floor, tables, and refrigerator handle), and the water fountain near the restrooms. Wipe samples were taken from the hands of 12 employees from the cathode ray tubes processing area and other areas before they left work, using wipes from the SKC Full Disclosure colorimetric test kit. This test kit identifies lead on surface wipe samples through a color change process and has a visual identification limit of 18 *μ*g of lead. The hands of eight of 12 employees tested positive for lead, even though they had washed their hands with soap and water before testing. NIOSH also took a wipe sample from uniforms of employees’ front shoulder area. Twelve of 13 uniforms tested positive for lead.

NIOSH investigators noted that the local exhaust ventilation system at the cathode ray tubes crushing operation recirculated potentially contaminated air back into the production area. There were no showers in facility A, and employees used brooms to sweep the work area, creating airborne dust. Because the changing area for employees who broke cathode ray tubes was not adjacent to the cathode ray tubes work area, employees could track lead-containing dust through the facility. Personal items, food, and work clothing and equipment were stored together in the changing area. All findings from the NIOSH health hazard evaluation were communicated to the employer and employees, along with recommendations to reduce exposure.

## Discussion

The U.S. Congress passed the Workers’ Family Protection Act in 1992 (6). The Act requires NIOSH to study take-home exposure of hazardous chemicals and substances, including lead. NIOSH found evidence that take-home exposure is a widespread problem (6). Workplace measures found to be effective in preventing take-home exposures included 1) reducing exposure in the workplace using the hierarchy of controls*; 2) changing clothes and shoes before going home and leaving soiled clothing at work for laundering; 3) storing street clothes in separate areas of the workplace to prevent contamination; 4) showering before leaving work; and 5) prohibiting removal of toxic substances or contaminated items from the workplace. NIOSH noted that preventing take-home exposure is key because decontaminating homes and vehicles is not always effective in the long term. Normal house cleaning and laundry methods are inadequate, and decontamination can potentially lead to hazardous exposures among those workers performing the cleaning activities.

CDC considers a BLL of 5 *μ*g/dL as the upper level of the reference range in children at which public health actions should be initiated (7). The National Toxicology Program found sufficient evidence that BLLs <5 *μ*g/dL in children are associated with attention-related behavioral problems and decreased cognitive performance (indicated by lower academic achievement, lower intelligence quotient, and decreases in certain cognitive measures) (8). There is limited evidence that BLLs <5 *μ*g/dL are associated with delayed puberty and reduced kidney function in children aged ≥12 years (8). There is sufficient evidence that BLLs <10 *μ*g/dL are associated with delayed puberty and decreased postnatal growth, and limited evidence that BLLs <10 *μ*g/dL are associated with increased serum immunoglobulin E and allergy diagnosed by skin prick testing (8). The NIOSH Adult Blood Lead Epidemiology and Surveillance System uses a surveillance case definition for an elevated BLL in adults as ≥10 *μ*g/dL. The National Toxicology Program found sufficient evidence that BLLs <5 *μ*g/dL in adults is associated with decreased glomerular filtration rate and reduced fetal growth in pregnant women (8). There is sufficient evidence that BLLs <10 *μ*g/dL in adults are associated with increased incidence of essential tremor, increased blood pressure, increased risk for hypertension, and increased risk for spontaneous abortion and preterm birth (8). There is limited evidence that BLLs <10 *μ*g/dL in adults are associated with psychiatric effects, decreased hearing, decreased cognitive function, increased incidence of amyotrophic lateral sclerosis, increased cardiovascular mortality, and electrocardiography abnormalities (8). However, current occupational exposure levels are not protective of workers.†

The investigation of lead poisoning includes examining common sources of lead exposure, such as deteriorating lead paint, as well as other sources when investigation of the home does not suggest a source. With the increasing use of electronic devices and subsequent disposal and recycling of those devices, exposure to substances such as lead contained within the devices is an emerging occupational health concern in the e-scrap industry. The patchwork of state regulations overseeing e-scrap recycling in the United States addresses possible damage to the environment, but health-based regulations are lacking. Approximately 130 million metric tons of e-scrap were recycled in the United States in 2010 (9), and this scrap stream contains many types of toxicants (cadmium, polybrominated diphenyl ethers, polychlorinated biphenyls, and polycyclic aromatic hydrocarbons) that are not routinely screened for in adult workers or children. The cases described in this report were uncovered through routine lead screening, but other undetected chemicals might also be coming home from e-scrap worksites. Pediatric health care providers should query parents about their occupations and to assess the risk for exposure to various substances found in occupational settings.

**Summary**

What is already known on this topic?

Lead is a neurodevelopmental toxicant, and no safe blood lead level (BLL) in children has been identified. Parental occupational take-home exposures are a source of lead exposure in children.

What is added by this report?

This report describes a novel source of take-home exposure from a parent who worked in a facility that recycled used electronics (e-scrap).

What are the implications for public health practice?

When evaluating children with elevated BLLs, public health professionals and clinicians should inquire about parental occupations because of the implications of take-home exposure. E-scrap recycling is an emerging area of concern as a source of occupational exposures among workers and a source of take-home exposures.

## Footnotes

* The first step in the hierarchy is eliminating or substituting hazardous processes or materials, which reduces hazards and protects employees more effectively than other approaches. Prevention through design (e.g., considering elimination or substitution when designing or developing a project) reduces the need for additional controls in the future. The second step is engineering controls, which reduce employees’ exposures by removing the hazard from the process or by placing a barrier between the hazard and the employee. Engineering controls protect employees effectively without placing primary responsibility of implementation on the employee. The third step is administrative controls, which refers to employer-dictated work practices and policies to reduce or prevent hazardous exposures. Their effectiveness depends on employer commitment and employee acceptance. Regular monitoring and reinforcement are necessary to ensure that policies and procedures are followed consistently. The last option is personal protective equipment (PPE) because it is the least effective means for controlling hazardous exposures. Proper use of PPE requires a comprehensive program and a high level of employee involvement and commitment. The right PPE must be chosen for each hazard. Supporting programs such as training, change-out schedules, and medical assessment might be needed. PPE should not be the sole method for controlling hazardous exposures; rather, PPE should be used until effective engineering and administrative controls are in place.

† In the United States, employers in general industry are required by law to follow the Occupational Safety and Health Administration lead standard (29 CFR1910.1025). This standard was established in 1978 and has not yet been updated to reflect the current scientific knowledge regarding the health effects of lead exposure. Under this standard, the permissible exposure limit (PEL) for airborne exposure to lead is 50 micrograms per cubic meter of air (*μ*g/m^3^) for an 8-hour time-weighted average (TWA). The standard requires lowering the PEL for shifts that exceed 8 hours, medical monitoring for employees exposed to airborne lead at or above the action level of 30 *μ*g/m^3^ (8-hour TWA), medical removal of employees whose average BLL is ≥50 *μ*g/dL, and economic protection for medically removed workers. Medically removed workers cannot return to jobs involving lead exposure until their BLL is <40 *μ*g/dL. The PEL might prevent overt symptoms of lead poisoning but does not protect workers from lead’s contributions to conditions such as hypertension, renal dysfunction, reproductive effects, and cognitive effects.
